# Bio‐Inspired Motion Mechanisms: Computational Design and Material Programming of Self‐Adjusting 4D‐Printed Wearable Systems

**DOI:** 10.1002/advs.202100411

**Published:** 2021-05-14

**Authors:** Tiffany Cheng, Marc Thielen, Simon Poppinga, Yasaman Tahouni, Dylan Wood, Thorsten Steinberg, Achim Menges, Thomas Speck

**Affiliations:** ^1^ Institute for Computational Design and Construction (ICD) University of Stuttgart Keplerstraße 11 Stuttgart 70174 Germany; ^2^ Cluster of Excellence *IntCDC* University of Stuttgart Keplerstraße 11 Stuttgart 70174 Germany; ^3^ Plant Biomechanics Group, Botanic Garden University of Freiburg Schänzlestraße 1 Freiburg 79104 Germany; ^4^ Freiburg Materials Research Center (FMF) University of Freiburg Stefan‐Meier‐Straße 21 Freiburg 79104 Germany; ^5^ Division of Oral Biotechnology, Center for Dental Medicine, Medical Center, Faculty of Medicine University of Freiburg Hugstetterstraße 55 Freiburg 79106 Germany; ^6^ Cluster of Excellence *liv*MatS @ FIT University of Freiburg Georges‐Köhler‐Allee 105 Freiburg 79110 Germany

**Keywords:** adaptive structures, additive manufacturing, biomimetics, digital fabrication, material computation, self‐shaping material systems

## Abstract

This paper presents a material programming approach for designing 4D‐printed self‐shaping material systems based on biological role models. Plants have inspired numerous adaptive systems that move without using any operating energy; however, these systems are typically designed and fabricated in the form of simplified bilayers. This work introduces computational design methods for 4D‐printing bio‐inspired behaviors with compounded mechanisms. To emulate the anisotropic arrangement of motile plant structures, material systems are tailored at the mesoscale using extrusion‐based 3D‐printing. The methodology is demonstrated by transferring the principle of force generation by a twining plant (*Dioscorea bulbifera*) to the application of a self‐tightening splint. Through the tensioning of its stem helix, *D. bulbifera* exhibits a squeezing force on its support to provide stability against gravity. The functional strategies of *D. bulbifera* are abstracted and translated to customized 4D‐printed material systems. The squeezing forces of these bio‐inspired motion mechanisms are then evaluated. Finally, the function of self‐tightening is prototyped in a wrist‐forearm splint—a common orthotic device for alignment. The presented approach enables the transfer of novel and expanded biomimetic design strategies to 4D‐printed motion mechanisms, further opening the design space to new types of adaptive creations for wearable assistive technologies and beyond.

## Graphical abstract

This paper presents a design and material programming approach for adaptive material systems through a case study of biomimetic design. The twining air potato (*Dioscorea bulbifera*) provides stability against gravity by tensioning its stem helix and generating a squeezing force. Based on *D. bulbifera*'s force generation, 4D‐printing is employed to produce adaptive wearable systems with passive, targeted shape changes.

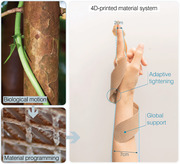

## 1 Introduction

Plants perform a variety of functionally robust and often reversible motions. Passive‐nastic movements are particularly promising for biomimetic approaches, as they are structurally predetermined and function without additional investment of metabolic energy from the plant. This is due to the fact that the respective motile plant structures often show hygroscopic behavior in their individual cells and tissues, which can absorb and desorb water molecules from the environment. Individual deformation on the cellular level takes place perpendicular to the orientation of cellulose microfibrils, causing anisotropic swelling or shrinking. When arranged in the form of a functional bilayer, the differential expansion between the individual cells of a motile plant organ's structuring results in various global shape changes.^[^ ^1^, ^2^ ^]^

The hygroscopic working principles of motile plant structures have, in an abstracted way, inspired numerous adaptive structures that do not rely on elaborate technical equipment nor require the supply of operating energy.^[^ ^3^ ^]^ In particular, the bending behavior of conifer cones^[^ ^4^ ^]^ has been emulated in bilayers crafted from various materials, ranging from cellulosic paper and polymer composites^[^ ^5^, ^6^, ^7^ ^]^ to wood veneer and epoxy‐bonded glass fiber composites.^[^ ^8^, ^9^ ^]^ Similar bio‐inspired systems for self‐shaping have even been upscaled for building elements and construction‐scale applications.^[^ ^10^, ^11^, ^12^ ^]^ Besides the opening and closing of conifer cones, other behaviors have also been replicated. Controlled bending or twisting in structures can be achieved through several techniques, including stretching thin sheets to form a residually stressed bilayer^[^ ^13^ ^]^ or by magnetically orientating particles within the bilayer's microstructure.^[^ ^14^, ^15^ ^]^

4D‐printing, the additive manufacturing technique for producing programmable matter which can reshape itself or change its behavior over time,^[^ ^16^ ^]^ has emerged as an automated method for embedding anisotropy in structured material systems. Smart materials with stimuli responsiveness have been 4D‐printed in multiple approaches^[^ ^17^, ^18^, ^19^, ^20^, ^21^, ^22^, ^23^ ^]^; however, both the materials as well as equipment that can process them are often custom‐made or highly specialized, making these techniques less affordable in both research and practice. While more accessible desktop 3D‐printers have the capacity to create reversible shape changes with hygroscopically actuated materials,^[^ ^24^, ^25^, ^26^ ^]^ these systems are typically designed and fabricated in the form of a simplified bilayer. 4D‐printing the differentiated material organization and multifunctionality of motile plant structures necessitates a design‐oriented approach in material programming.

## 2 Material Programming with 4D‐Printing

Computational design has the potential to transfer complex and compounded behaviors of different plant role models to promising technical systems. We present a material programming approach for 4D‐printing bio‐inspired material systems and describe the key computational methods for structuring motion mechanisms at the mesoscale.^[^ ^27^ ^]^ This includes the modularization of self‐shaping material systems as assemblies of sub‐mechanisms with custom‐prescribed properties and behaviors. This modular architecture makes it possible to manage the integration of multiple motion mechanisms within one material system. We explain the details of designing motion mechanisms with customized anisotropies and magnitudes of hygroscopic actuation, which can be printed by a standard fused filament fabrication (FFF) 3D‐printer utilizing various types of commercially available filament materials.

**Figure 1:**
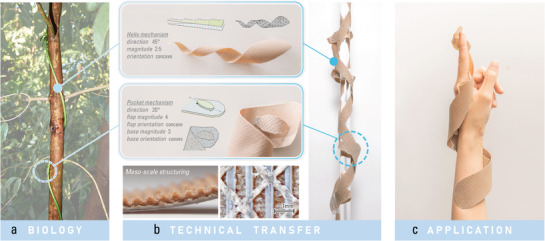
The biomimetic process as used in this paper, from role model to wearable material system: a) the investigation and understanding of *Dioscorea bulbifera*'s biomechanics and functional morphology for generating forces by twining around a support and expanding its stipules;^[^ ^28^ ^]^ b) the abstraction of these functional principles and their transfer to bio‐inspired motion mechanisms through computational design methods; c) the combining and stacking of several motion mechanisms as one multifunctional 4D‐printed material system.

Our methodology is demonstrated through a case study of biomimetic design (**Figure** **1**). The twining air potato (*Dioscorea bulbifera*) exhibits a squeezing force to its support in order to provide stability against gravity. Based on this strategy of force generation,^[^ ^28^ ^]^ we transfer the technical principles to a prototype of a user‐customized, self‐tightening orthotic splint. We first outline our biomimetic process, using a bottom‐up (biology push) approach,^[^ ^29^ ^]^ starting from the selection of *D. bulbifera* as a role model to the abstraction and translation of its functional principles as 4D‐printed motion mechanisms that can adapt to moisture stimuli. We then 4D‐print the holistic material system using wood‐filled filaments and evaluate several variations by measuring their squeezing forces under controlled settings. As a use case, we finally develop a first working prototype of a wrist‐forearm splint, a common orthotic device.

## 3 Biomimetic Process

### 3.1 Selecting a Suitable Role Model

In this paper, we have used orthotic devices as a motivating example, as there is immense potential for passive adaptivity in wearable assistive technologies for medical and sports applications. Orthoses and prostheses fall in the category of externally applied devices used to modify the structural and functional characteristics of the neuromuscular and skeletal systems or replace (either wholly or in part) an absent or deficient limb segment, respectively.^[^ ^30^ ^]^ Moreover, orthotic and prosthetic devices must be properly fitted in order to provide support, and these devices often need to be refitted during their course of use. For example, muscular atrophy commonly occurs from injury‐related immobilization with an orthotic cast, leading to the necessity for frequent adjustments to prevent slipping.

Velcro^[^ ^31^ ^]^ is a well‐known and widespread biomimetic fastening mechanism often employed in orthoses. Inspired by the hook‐covered fruits of burdocks (*Arctium* sp.) and avens (*Geum* sp.), this hook and loop system can be adjusted by opening, readjusting, and closing it again. Although Velcro‐equipped devices eliminate the need for frequent refitting appointments with a medical practitioner, readjusting the fastening system still requires full detachment of the strips. Without a mechanism for incremental changes and needing to completely release the pressure at every adjustment attempt, it may be cumbersome and challenging for the patient to make the correct adjustments, risking constriction and pain from improper fit.^[^ ^32^ ^]^

Twining plants are prime examples of adaptive biological structures. By winding around their host plants via growth‐based motion sequences, they are able to climb up to dozens of meters; some species even reach lengths of more than 100 m.^[^ ^33^, ^34^ ^]^ Interestingly, *D. bulbifera* exhibits a squeezing force on the host plant through the tensioning of its stem helix, which has been shown to occur with the expansion of stipules at its leaf base.^[^ ^28^ ^]^ The quality of this squeezing behavior is smooth and continuous (from loose to tight), rather than discrete (either fully detached or attached) when compared to the Velcro system—presenting a promising opportunity for incremental adjustment in adaptive, wearable devices.

### 3.2 Abstraction of Biomechanics and Functional Morphology

The above‐mentioned squeezing behavior of *D. bulbifera* provides stability against slipping due to gravity, allowing the plant to ascend smooth supports. The procedure for generating squeezing forces can be generally described in two phases: 1) the loose wrapping of a helix around an existing support, and 2) the delayed expanding of discrete lateral structures (distributed on the inner surface of this helix) which apply pressure to the support— thus pushing the helix outward, and thereby tensioning the system.^[^ ^28^ ^]^

**Figure 2:**
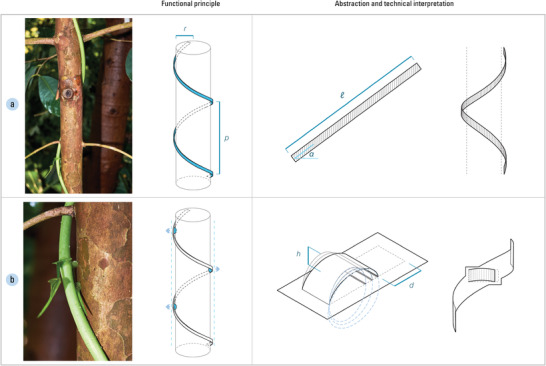
*Dioscorea bulbifera* is characterized by a twining stem with discrete stipules that expand to produce a gap between the stem and its support, thus putting the stem under tension: a) first, the twining stem loosely winds around its support, a behavior abstracted as a twisting strip called the helix mechanism which coils around a support structure; b) then, stipules grow at the petiole bases, abstracted as hinged flaps called pocket mechanisms that curl to create space between themselves and the support structure at distributed points. Consequently, the pocket mechanisms push the helix mechanism outward, tensioning the helix and generating squeezing forces. These motion mechanisms are integrated into one material system comprising multiple functions.

We translate these functional principles as the helix mechanism and the pocket mechanism. The helix mechanism is essentially a long strip of length ℓ which bends at angle *α*, resulting in a radius of curvature r and allowing it to wind around a support structure (**Figure** **2**a). As the bending angle determines how deep or shallow the twist is, it (along with the magnitude of bending) also determines the pitch p of the helix (cf. ^[^ ^13^, ^35^ ^]^). The pocket mechanism mimics the stipule growth that is responsible for creating tension. Interpreted as hinged flaps that sit on top of the helix, they curl to create space between their twisting base surface and the support structure (Figure 2b). The depth h created between the base surface and support is determined by the length and bending magnitude of the hinged flap (subsequently, the travel distance d of the flap can also be known). We then transfer and implement these two technical interpretations of *D. bulbifera*'s force generation to 4D‐printed motion mechanisms through computational design.

## 4 Technical Transfer

### 4.1 Designing and Programming Mesoscale Material Structures

We represent self‐shaping material systems as compounded assemblies of simpler sub‐mechanisms; these basic units for shape change are called motion mechanisms. A single motion mechanism is composed of at least two different materials: a stimuli‐responsive actuating material and a restricting material, which remains relatively stable (in comparison with the former). Upon stimuli response, the two materials together result in a differential volume change, producing spatial transformations through bending.^[^ ^36^ ^]^ While the bending behavior of a motion mechanism is primarily governed by its material constituents, their properties can be further tuned by structuring the material at the mesoscale.

The motion mechanism's shape transformation is defined by its bending direction, orientation, and magnitude. The direction of bending in a motion mechanism is determined by the anisotropy of the actuating material. Paths of material deposition during fabrication will bias bend according to the predominant angle; the restricting material is extruded in the same direction as bending, while the actuating material is aligned perpendicular to it. The orientation of bending is determined by the spatial ordering of the actuating and restricting materials; the order in which the materials are layered will create either a convex or concave transformation. The magnitude of bending is determined by the overall flexural rigidity of the motion mechanism, as well as the actuating material's capacity for expansion or contraction. Programming parameters that influence flexural rigidity include the thickness and porosity of each material. Flexural rigidity can be increased by simply printing more layers to add thickness. On one hand, thicker layers of the actuating material will decrease the speed of self‐shaping, as moisture would need to penetrate a larger volume and therefore require a longer duration of diffusion. On the other hand, the ratio between actuating to restricting material can also be adjusted; thus, porosity can be used to affect flexural rigidity without changing thicknesses. Higher porosity, achieved with larger spacing between material paths, lowers the flexural rigidity while requiring less time to self‐shape (see Digital Fabrication in Section 7 for more details).

**Figure 3:**
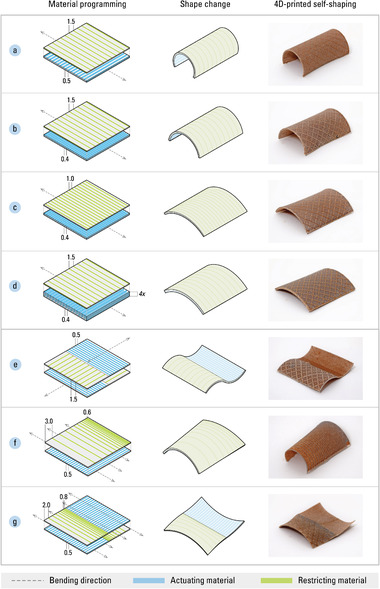
A motion mechanism can produce a breadth of shape changes through the programming of both the actuating material (shown in blue) and restricting material (shown in green). The following shows how differences in extrusion paths result in varying self‐shaping behaviors: a) the actuating material paths are spaced at 0.5 mm, and the restricting material paths at 1.5 mm; b) the actuating material paths are spaced at 0.4 mm, while the restricting material paths remain at 1.5 mm (resulting in less bending); c) the actuating material paths are spaced at 0.4 mm, and the restricting material paths are more densely packed with 1.0 mm offsets (resulting in even less bending); d) the actuating material maths are spaced at 0.5 mm in multiple layers, while the restricting material paths remain at 1.5 mm in a thin layer (resulting in slower actuation, in addition to decreased bending). Multiple motion mechanisms can be aggregated to transform into more nuanced geometries: e) motion mechanisms with identical bending directions but opposite bending orientations (resulting in a wavy surface); f) discrete motion mechanisms forming a gradation of bending magnitudes (resulting in a surface with decaying actuation); g) two motion mechanisms with parallel bending directions and flipped bending orientations (resulting in an anticlastic surface).

We developed a parametric model for programming the motion mechanisms and converting their mesoscale design to 3D‐printable machine instructions. Tuning these parameters for the actuating and restricting materials in different combinations will produce a large variety of motion mechanisms from initially flat states (**Figure** **3**a–d). We use the bottom‐up, forward modeling approach^[^ ^37^, ^38^ ^]^ to combine multiple motion mechanisms into higher‐level assemblies, creating a rich array of more complex movements (Figure 3e–g).

### 4.2 Fabricating Customized Motion Mechanisms

We then 4D‐print the helix and pocket mechanisms, abstracted from *D. bulbifera*, as the two main types of motion mechanisms in our self‐shaping material system. In order to understand the parameters for mesoscale material structuring and establish the variables for achieving targeted shape changes, we conduct a parameter study for each mechanism. The control parameters for creating variations in shape are material‐specific, and we perform the study using a moisture‐reactive filament for the actuating material and a relatively stable thermoplastic filament for the restricting material. The resultant swelling and shrinking of the actuating material in response to changes in relative humidity (RH) causes the motion mechanism to bend.

#### 4.2.1 Bio‐Inspired Helix Mechanism

**Figure 4:**
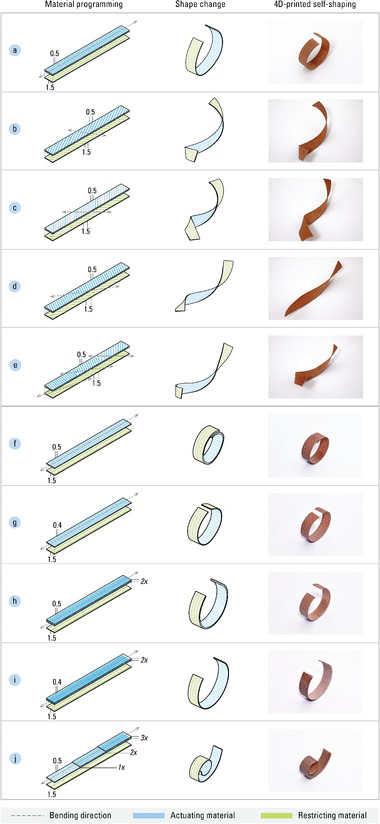
The properties of the helix mechanism are programmed by tailoring the mesoscale structure. The following shows how varying the angle of extrusion paths result in 4D‐printed strips with different bending directions: a) the helix mechanism bending at 0°; b) bending at 30°; c) bending at 45°; d) bending at 60°; e) and with a gradient of bending directions, from 15° to 45°. In addition to bending direction, the bending magnitude of a helix mechanism can be customized by tuning porosity at the mesoscale: f) extrusion paths with wide offsets result in high porosity (leading to increased bending); g) more densely packed extrusion paths result in less porosity (leading to decreased bending); h) decreased bending is also achieved through thicker layers, shown here with widely spaced extrusion paths; i) and with densely packed extrusion paths. j) Finally, the helix mechanism can be programmed with varying magnitudes of bending, forming a spiral.

The helix mechanisms are implemented as strips that twist in the direction perpendicular to the anisotropic paths of the actuating material. The strip length of the helix mechanism, along with the angle and amount of twisting, defines its coiling around a cylindrical structure at a specific pitch and radius. Through both kinematic models and empirical 4D‐printed specimens, we identified the role of each parameter and determined the relationships between anisotropy and bending direction (**Figure** **4**a–e), as well as flexural rigidity and bending radius (Figure 4f–j).

#### 4.2.2 Bio‐Inspired Pocket Mechanism

**Figure 5:**
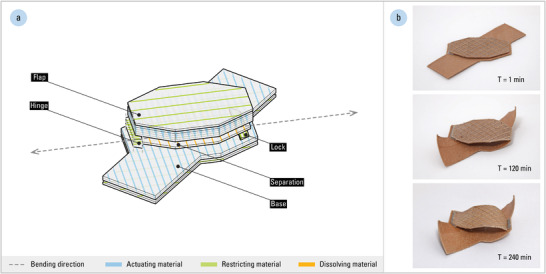
The pocket mechanism is an assembly of five components, each programmed to perform a different function: a) the flap and base share the same bending direction; however, their bending orientations are inverse to each other. The flap is also thicker than the base, resulting in less porosity and causing slower actuation. b) Consequently, the pocket mechanism's base (i.e., the helix mechanism) first actuates, then the flap slowly follows, self‐shaping until it passes over the directional lock.

The pocket mechanisms are implemented as space‐making flaps which are stacked onto the helix mechanism. To produce the pocket mechanism, we combine several motion mechanisms in a single fabrication process (**Figure** **5**). The pocket mechanism comprises the following: a curling flap on top of a base helix, connected to each other only at one end by a compliant hinge. A directional lock allows the free tip of the flap to travel across it in one direction but not the other. As these components are printed in one fabrication sequence, a sacrificial separation layer exists to prevent the flap from adhering to its base during the heated extrusion process.

Each of these five components can be programmed individually. To create a pocket of space, the flap and base must have opposing bending orientations, but with identical bending directions to prevent them from clashing while changing shape. Their bending magnitudes and actuation times can be differentiated from each other. The hinged flap is programmed to have a lower porosity than that of its base surface, in order to delay the pocket mechanism's moisture‐induced actuation until after the initial coiling of the helix mechanism.

As the hinge and lock do not self‐shape, both are composed of only the restricting material and without any actuating material. The hinge is programmed to be compliant, allowing the flap and base to change shape independently. The lock geometry is designed as a slope, which facilitates the free tip of the flap to travel over it in one direction when curling, while preventing the flap from collapsing when loaded. Determining the flap's displacement additionally allows us to strategically place the directional lock; several locks can be added to create resistance to pressure at multiple steps.

The separation layer, composed of a dissolvable filament, is a removable support structure used to isolate the flap from the base during fabrication. After fabrication, the entire 4D‐printed material system is placed in a solvent for softening and dissolving the separation layer, creating a gap between the flap and base. As an imprint of its mesostructured surface is left at the interfaces of the two functional areas even after removal, the separation layer is programmed with an anisotropy that compliments that of both the flap and base.

### 4.3 Evaluation of the 4D‐Printed Material System

The helix and pocket mechanisms are aggregated to form functional self‐shaping material systems with bio‐inspired tensioning. To assess the performance of the 4D‐printed behavior, we compare two material systems with and without the pocket mechanisms. Both systems comprise the same set of double helices with mirrored chirality (one left‐handed and one right‐handed); however, one system also contains pocket mechanisms. The system's ability to self‐stabilize and generate force is evaluated in two experiments.

#### 4.3.1 Self‐stabilization

**Figure 6:**
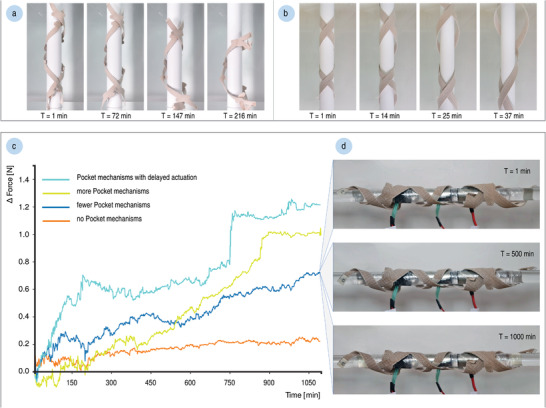
The 4D‐printed helical material system with the bio‐inspired tensioning mechanism was able to self‐stabilize on supports with diameters as small as 15 mm: a) shown here are time‐lapse stills of the initially unsaturated (dry) bio‐inspired material system with pocket mechanisms releasing from a 30 mm support during an elapsed period of 216 min as it reaches full moisture saturation; b) in comparison, after only 37 min, the helical material system without pocket mechanisms was destabilized from a support of the same size. c) The grip forces of initially saturated (wet) 4D‐printed helical material systems with various pocket types were measured, during separate experiments, on a 30 mm diameter support at exposure to low RH; these results compare the change in forces of the pure helix mechanism (0.22 N increase), with pocket mechanisms spaced apart at 220 mm along the helix (0.72 N increase), additional pocket mechanisms with 110 mm spacings (1.01 N increase), and the same number of pocket mechanisms with further actuation delay (1.24 N increase); d) shown here is the 4D‐printed material system, with pocket mechanisms spaced 220 mm apart, equipped with force sensors and self‐tightening in low RH.

We tested the ability of the 4D‐printed material system to stabilize itself on a smooth support. Two double helices with mirrored chirality can grip onto an upright, standing cylindrical structure without sliding down. However, a system consisting of the same helices but also containing the pocket mechanisms can stabilize itself on support structures of smaller diameters as well, expanding the range of diameters onto which it can grip. We demonstrated that our material system containing pocket mechanisms was able to adapt and grip onto supports of diameters ranging from 15 to 30 mm (**Figure** **6**a). In comparison, the same material system consisting of just the helices (without any pocket mechanisms) was only able to successfully stabilize itself on a 30 mm diameter support (Figure 6b).

#### 4.3.2 Force Generation

We measured the squeezing forces of the 4D‐printed material system. For each experiment, we produced two batches of 4D‐printed specimens: one batch containing a left‐handed helix mechanism paired with a right‐handed helix mechanism, and the second batch containing the same pair of helix mechanisms but combined with pocket mechanisms spaced apart at 220 mm along the helix. Additional sets of helical material systems with various pocket types were 4D‐printed, including helix mechanisms with more pocket mechanisms spaced apart at 110 mm and helix mechanisms with pocket mechanisms that were programmed with further actuation delay.

Our experiments simultaneously tracked the change in squeezing forces of the helix mechanism as well as the helix mechanism combined with pocket mechanisms, as they acclimate to a dry environment while gripping onto a 30 mm diameter support and form a tighter grip over time (Figure 6c). Compared to specimens comprising only the helix mechanism, the experiment showed that specimens containing the pocket mechanism (spaced apart at 220 mm) generated 327% higher force values. Specimens with more pocket mechanisms (spaced apart at 110 mm) exhibited even more force, 459% than the specimens without the pocket mechanism, as this causes greater distribution of forces across the same area. Finally, programming those additional pocket mechanisms with further actuation delay led to a 564% increase in force generation than the purely helical material system, demonstrating the most tensioning.

### 4.4 Toward a Self‐Adjusting Orthotic Device

**Figure 7:**
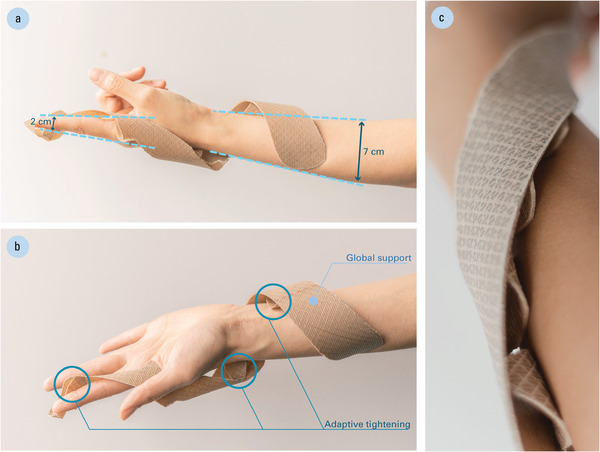
A first prototype of a common orthotic device, the wrist‐forearm splint, as an adaptive bio‐inspired functional mechanism: a) this helix mechanism is designed to wrap the body from forearm to finger; b) the helix mechanism serves as the primary structure and provides global support, while the pocket mechanisms allow the device to self‐tighten through several points of pressure; c) close‐up view of the many densely packed pocket mechanisms placed at the interface between the helix mechanism and skin.

As an outlook of a possible application, we transferred these bio‐inspired motion mechanisms to a prototype of a 4D‐printed orthotic splint with adaptive tightening (**Figure** **7**). Unlike the consistent diameter support structures that were used for the force measurements and initial parameter studies, the human body is quite varied in shapes and sizes even across one limb. To implement a self‐tightening wrist‐forearm splint, we employed a top‐down, inverse modeling approach to design the device for a specific user. In this process, the desired geometry (in its actuated state) was modeled on the physical arm, rather than by digitally programming the flat assembly.

**Figure 8:**
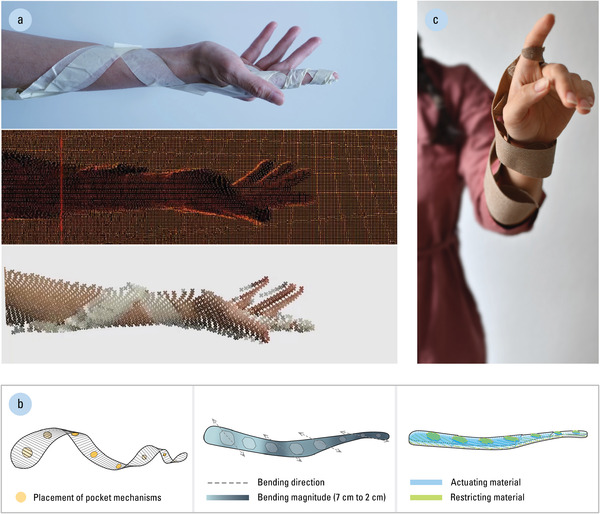
Programming a self‐adjusting orthotic splint, demonstrated through a top‐down, inverse modeling approach: a) the envisaged process begins with the physical modeling of a splint design on the patient's arm by a medical care specialist, which is then 3D‐scanned, allowing both the splint and arm geometries to be digitally reconstructed; b) the splint design is processed as a surface and analyzed, then unrolled with the target curvature information (bending directions, orientations, and magnitudes) assigned to each motion mechanism in the flat assembly, and finally the toolpaths are generated for fabrication; c) the adaptive 4D‐printed proof of concept is worn by a user.

The wearable device was designed directly on the body and can be mocked up with colored masking tape to represent a double helix mechanism squeezing from the forearm to fingers, with the placement of pocket mechanisms indicated in another color. The device can be designed to feature a multitude of pocket mechanism types (ranging from thin and shallow, to wide and deep) and be populated with any quantity of them (while meeting a minimum gap tolerance of 2 mm between each to avoid fusing together during the extrusion process). The entire arm was then 3D‐scanned (Kinect for Xbox One, Microsoft, Washington, USA), digitizing both the target geometry as well as the body part (**Figure** **8**a). The initial design was reconstructed as a developable surface for analyzing the principal curvatures and their radii. After extracting these values, the design can be unrolled as a flat, printable geometry; in order to produce the different curvature information (bending directions, orientations, and magnitudes), the flat geometry was discretized into an assembly of motion mechanisms with various programmed properties. Finally, the differentiated mesoscale material structure was converted into a set of toolpaths and G‐code instructions, which were directly executed by the machine (Figure 8b) and manufactured in the flat state.

Adaptive tensioning and force generation can provide promising benefits for implementation in devices that interact with the human body, using moisture from either the body or environment as a stimulus. We envision that wearable devices might be designed to release pressure and allow ventilation in reaction to fluctuations in moisture from body sweat, or loosen for temporary removal in daily activities such as showering when triggered by steam in the environment. During long‐term use, a self‐shaping device for immobilization can be programmed to slowly tighten itself over time (eliminating the need for frequent appointments to remove and reapply the cast as a result of muscle atrophy).

## 5 Discussion

We have developed a computational design method for physically programming self‐shaping systems with differentiated mesoscale material structuring. This material programming approach allows individual motion mechanisms, each containing different specifications for shape change, to be aggregated as one 4D‐printed material system.

We first performed a parameter study on tailoring motion mechanisms using extrusion‐based 3D‐printing and identified the control parameters for a particular set of filaments. Material behaviors such as the anisotropy and magnitude of hygroscopic actuation can be customized by tailoring the mesostructure during 4D‐printing. Although we can achieve a delayed actuation by decreasing the mesoscale porosity, the precise control of timing and duration should be further studied and verified in future experiments. Pure and wood‐filled thermoplastics were used only as a proxy to show how different movements can be achieved; other material combinations can also be used with the presented techniques by calibrating the control parameters. As all materials have their own strengths and weaknesses, they should be curated for the task at hand. In this study, we have employed moisture as a stimulus; while this material system responds to a rather wide range of humidity levels, a material with sensitivity to higher humidity fluctuations may be needed in areas of frequent sweat. In other applications where there is more variability in body temperature, a material system that reacts instead to heat might be more suitable.

We demonstrated our approach by using a role model with an application as a case study. Inspired by *D. bulbifera*'s capacity to generating squeezing forces, we abstracted its functional principles as two types of motion mechanisms. Next, we 4D‐printed and tested the holistic material system, which can self‐tighten and adaptively generate forces. When comparing our 4D‐printed material system to the natural role model, both the 4D‐printed specimens and *D. bulbifera* generated relatively low squeezing forces; however, the number and types of tensioning pocket mechanisms on our helical material systems can be tuned. Computational design and 4D‐printing can enable bio‐inspired material systems to incorporate a much higher number of pocket mechanisms for increased tensioning, as the addition of these mechanisms generally produced higher squeezing forces and allowed the system to grip onto supports of various (and tighter) cross‐sections. Furthermore, the 4D‐printed material system can even be equipped without any pocket mechanisms if tensioning is undesired. As hygroscopic materials are commonly affected by decreased stiffness at high moisture content, we have designed and programmed our material system such that the tightening occurs during dry and low RH conditions. We expect that newly developed materials with higher stiffnesses can alleviate this limitation in applications where a certain target pressure is required (e.g., for full immobilization in high RH environments). At the same time, variations in stiffness could also be incorporated as a design feature.

Finally, a use case was illustrated for designing and prototyping a custom orthotic device for adaptive tightening. Orthotic devices must be properly fitted to provide support. Because muscular atrophy commonly occurs from injury‐related immobilization, these devices require frequent readjustments over time. Thus, our presented approach shows an exciting potential for applications in wearable systems that interact with the human body by, for example, self‐tightening to address muscular atrophy or self‐releasing to allow short‐term ventilation and removal.

Not only can 4D‐printed systems allow for adaptation during their course of use, but they can also be tailored to unique patient scenarios. Customization in wearable assistive technologies has been shown to offer improved fit and comfort over mass‐produced, off‐the‐shelf ones.^[^ ^32^ ^]^ Craft‐based techniques, such as plaster casting and molding, are the traditional means of producing custom‐made orthoses and prostheses.^[^ ^39^ ^]^ However, additive manufacturing offers rapid and affordable customization options for patients,^[^ ^40^ ^]^ not only in matching the device to a patient's anatomy but also incorporating functions to meet the needs of treating a particular pathology. Attempts to transition toward mass‐customization processes face challenges in their applications in the industry, as digital design and modeling techniques are highly specialized.^[^ ^41^ ^]^

Through proving this concept with a first working prototype of a common orthotic device, we envision that this design process might enable medical experts to physically design, prototype, and custom‐fit self‐adjusting orthotic devices without any specialized knowledge in digital modeling.^[^ ^42^ ^]^ Furthermore, this approach extends the design space beyond the bio‐inspiration. As a result of computational design and additive manufacturing, our 4D‐printed splint can be modified to possess more desirable features (such as the pocket mechanisms with multiple tailored attributes) than occurring naturally in the plant role model—ultimately adding to the usefulness of bio‐inspired adaptive wearable devices.

## 6 Conclusion

We have demonstrated a material programming approach for designing and 4D‐printing bio‐inspired motion mechanisms that can adapt to environmental stimuli. As a showcase, we focused on *D. bulbifera*'s functional principles for tensioning and used orthotics as a motivating application. These motion mechanisms can be produced with any consumer desktop FFF 3D‐printer capable of reading and executing G‐code. Using computational design to control the anisotropic extrusion for a set of materials, we tailored self‐shaping material systems at mesoscale precision (alternative filament combinations can also be used). While we have exploited hygroscopy to present these methods, we hope that as more filaments with new modes of responsiveness become available, shape changes induced by stimuli other than moisture (such as heat or light) will be unlocked. The presented process has made it possible to mimic and apply the tensioning mechanism found in *D. bulbifera* to self‐adjusting wearable systems.

The modular architecture of our methodology can be used to create more complex configurations of self‐shaping structures, expanding the range of role models from biology that can be transferred to technical systems via 4D‐printing. To the best of our knowledge, the presented 4D‐printed motion mechanisms constitute the first examples of a building block approach to programming bio‐inspired adaptive systems, with potential applications extending beyond self‐adjusting wearable devices to self‐shaping architectural elements.^[^ ^43^ ^]^ The combination and stacking of several motion mechanisms with discrete movement kinematics (e.g., wrapping, squeezing), functions (e.g., alignment, stiffening), and sequencing (e.g., first twisting, and then generating force) into one material system opens up a powerful design space for a multitude of on‐demand printable solutions.

## 7 Experimental Section

##### Computational Design

Based on topological and geometrical constraints,^[^ ^38^, ^44^ ^]^ a toolkit was developed for designing mesoscale material structures^[^ ^45^ ^]^ through a visual programming environment (Grasshopper 3D) running within a computer‐aided design software (Rhinoceros 3D). As an extrusion‐based process, FFF 3D‐printing plays a dominant role in crafting features at the mesoscale; the paths of material deposition inherently create anisotropy. It is therefore essential to design the toolpath trajectory and quality of material extrusion. Controlled self‐shaping in a motion mechanism is enabled by multiple programming variables, which are assigned in the digital model for each material layer: anisotropy (angle from 0° to 180°), porosity (spacing from 0.4 mm to 0.6 mm for the actuating material, and 1.0 mm to 2.0 mm for the restricting material), layer height (from 0.10 mm to 0.25 mm), number of layers (1–6), speed of travel (700–1200 mm min^−1^), and flow of material (0.033 mm of filament per linear mm). These programming parameters can be input manually or, for more complex aggregations, modeled algorithmically. Before printing, the behavior of the motion mechanisms can also be visualized using a live physics engine (Kangaroo). The specified parameters were translated into the G‐code machine language (Voxel2GCode), which instructs the 3D‐printer on where to move, at what speeds, and with how much material. This fabrication data can be carried out by any FFF 3D‐printer capable of reading G‐code.

##### Digital Fabrication

A desktop FFF 3D‐printer with two print heads (FELIX Tec 4 Dual Head, FELIXprinters, Utrecht, Netherlands) was used for conducting this research. By following G‐code commands, the print head extrudes a selected material along planned paths with custom extrusion settings (Table S1, Supporting Information). A 3D‐printer with only one print head can also be used to replicate these results; however, this requires manual filament changes in between print pauses, as was done when printing the separation layer (using a third material). Materials were extruded using 0.35 mm diameter brass nozzles, with cartesian movements at 0.05 mm precision. The nozzle size, in combination with the material flow (which had been kept constant for this study), produced extruded paths with widths of approximately 0.5 ± 0.075 mm. This dimension, together with the value assigned for porosity, determines the actual pore size resulting between material depositions. Based on the fabrication setup, an interface or overlap was created between material paths with spacing values ⩽0.6 mm. With a spacing value of 0.7 mm, there becomes limited engagement between adjacent paths and little shape change was observed.

##### Materials

This approach can be applied to any two filament materials with different expansion coefficients, using various options already on the market. A combination of wood‐polymer composite (WPC) and acrylonitrile butadiene styrene (ABS) filaments was used. Whereas the ABS used for the restricting material (ABS, MakerBot Industries, New York, USA) was pure polymer, the WPC used for the actuating material (Laywoo‐D3 & LAYWOODmeta5, Lay Filaments, Cologne, Germany) contained 40% recycled wood fillers within a polymer matrix. Because of its hygroscopy, the WPC released moisture in dry or low RH conditions (Movies S1 and S2, Supporting Information) and gained moisture in wet or high RH conditions (Movies S3 and S4, Supporting Information). High‐impact polystyrene (HiPS) was used as a dissolvable filament, which had similar print settings as ABS. The separation layers were printed with HiPS (Smartfil SUPPORT, SmartMaterials3D, Alcalá la Real, Spain) and dissolved in D‐Limonene (Filament2Print, Pontevedra, Spain). The actuating material was extruded at a constant 190 °C, while both the restricting and dissolving materials were extruded at 200 °C. The materials were printed on a bed heated to 45 °C. All filaments were 1.75 mm diameter and housed in, as well as printed from, a dry storage box (PolyBox Edition II, Polymaker, Shanghai, China) maintained at 10–15% RH. This minimized any unwanted shape changes during production and ensured consistent performance.

##### Controlled Actuation

To study the effect of individual control parameters on a motion mechanism, 4D‐printed specimens were actuated under controlled conditions and their shape transformations were documented from wet to dry. All specimens were first primed in a water bath for at least 30 min to completely saturate the actuating material with moisture; then, after wiping excess water from the surface, they were relocated to a climate‐controlled dry environment (25–28% RH) for 6 h. The dimensions, weight, and curvature of all specimens were recorded immediately after removal from the print bed, at full saturation, and after drying (Table S2, Supporting Information). For each parameter studied, a minimum of three identical specimens was 4D‐printed and their results averaged. The climate‐controlled chamber included a glass display case (400 mm × 400 mm × 400 mm) which sat on top of a platform containing a humidity control device (MiniOne Humidity Generator, Preservatech, Bydgoszcz, Poland). An onboard computer (Raspberry Pi 3 Model B, Raspberry Pi Foundation, Cambridge, United Kingdom) and image input device (Raspberry Pi Camera v2, Raspberry Pi Foundation, Cambridge, United Kingdom) captured a photo of the 4D‐printed specimen every 60 s.

##### Self‐Stabilization Test

To assess the ability of this bio‐inspired material system to resist sliding on smooth cylindrical supports, 4D‐printed specimens were tested on vertical support structures from dry to wet conditions. Each specimen was wrapped around an upright, standing support structure, and left to equalize in ambient dry conditions (30% RH) for at least 24 h. The initially dry, tightly wrapping system was then transferred (still in the upright position) to a water tank (400 mm × 400 mm × 400 mm) until its grip loosened under direct exposure to water and finally slid down. Several diameters (15 mm, 20 mm, and 30 mm) were used, and each specimen was 4D‐printed twice. Via time‐lapse video (Nikon D3000 DSLR camera with digiCamControl software) at 30 s intervals, the experiment captured how long it took each specimen to become destabilized.

##### Force Measurements

The squeezing forces of two 4D‐printed specimens were evaluated at the same time. The setup of this experiment included a pair of same‐sized horizontal supports (Plexiglas tubes with diameters of either 30 mm, 50 mm, or 70 mm). Each support was equipped with three sensors for measuring three areas of the same specimen. Instead of load cells (cf. ^[^ ^28^, ^46^ ^]^), Interlink Force‐Sensitive Resistors (FSR 406) were employed to detect force; this sensor technology is more flexible in construction for placement on cylindrical substrates. The sensor‐equipped supports were wrapped in clear, polyethylene film to protect the sensors from large fluctuations in temperature and humidity and prevent noise in data. All specimens were primed by submerging in water for at least 30 min, wiped from excess water on the surface, and coiled around their support structures. Starting from their fully saturated conditions with a loose grip, the entire setup was exposed to a climate‐controlled dry environment (25–28% RH), slowly forming a tight grip within 18 h, after which the forces plateau over the course of 70 h (Figure S1, Supporting Information). The forces of each sensor, along with the temperature and humidity values (DHT22), were logged by a microcontroller (Arduino Uno, Arduino LLC, Torino, Italy) every 60 s during the experiment. The overall squeezing forces of several sized 4D‐printed specimens were recorded. For each size, this procedure was repeated for two cycles.

## Conflict of Interest

The authors declare no conflict of interest.

## Supporting information

Supporting Information

Supporting Information

Supporting Information

Supporting Information

Supporting Information

Supporting Information

Supporting Information

## Data Availability

The data that supports the findings of this study are available in the supplementary material of this article.
