# Supplementary material for: Bio‐Inspired Motion Mechanisms: Computational Design and Material Programming of Self‐Adjusting 4D‐Printed Wearable Systems
Source: Adv Sci (Weinh). 2021 May 14;8(13):2100411. doi: 10.1002/advs.202100411 (PMC8261511; doi:10.1002/advs.202100411)
Supplement: Supplementary file 1 — Supporting Information [file ADVS-8-2100411-s001.pdf]

© 2021 Wiley-VCH GmbH

ADVANCED SCIENCE
Open Access

Supporting Information

for *Adv. Sci.,* DOI: 10.1002/advs.202100411

Bio-inspired Motion Mechanisms: Computational Design and Material Programming of Self-adjusting 4D-printed Wearable Systems

Tiffany Cheng, Marc Thielen, Simon Poppinga, Yasaman Tahouni, Dylan Wood, Thorsten Steinberg, Achim Menges*, and Thomas Speck*

Supporting Information

Bio-inspired Motion Mechanisms: Computational Design and Material Programming of Self-adjusting 4D-printed Wearable Systems

Tiffany Cheng, Marc Thielen, Simon Poppinga, Yasaman Tahouni, Dylan Wood, Thorsten Steinberg, Achim Menges*, and Thomas Speck*

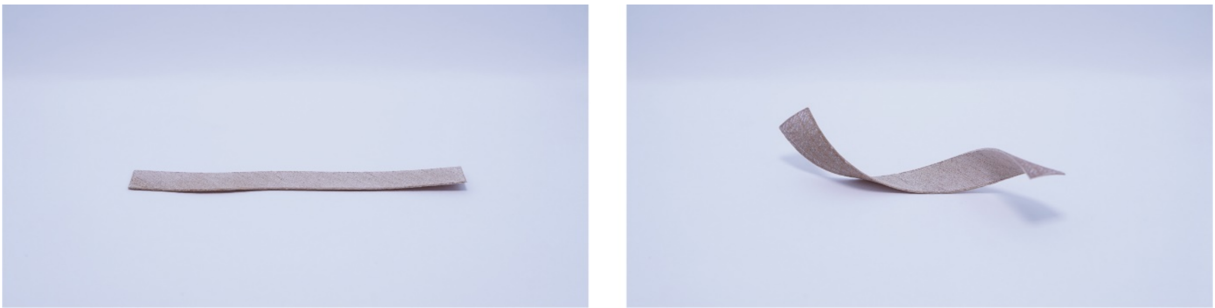

Movie S1. A 4D-printed helix mechanism equalizing in a climate-controlled chamber programmed at 25-28% RH (video playback at 1500x speed).

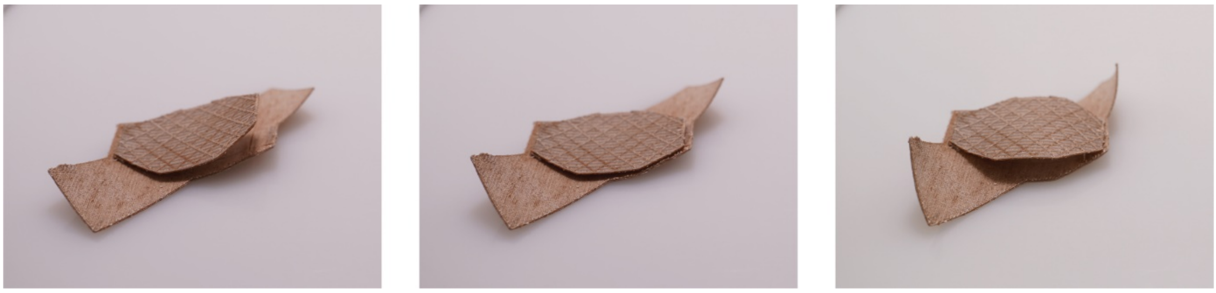

Movie S2. A 4D-printed pocket mechanism equalizing in a climate-controlled chamber programmed at 25-28% RH. The base is first to actuate, then the flap follows (video playback at 1500x speed).

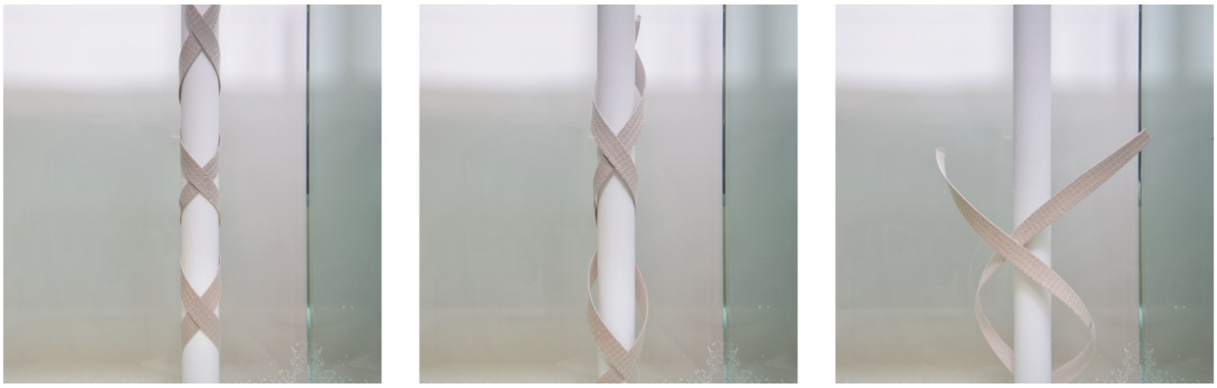

Movie S3. A 4D-printed helical material system is initially stabilized on a 3 cm diameter support structure. After direct exposure to water, the system self-releases from its support and no longer resists sliding (video playback at 500x speed).

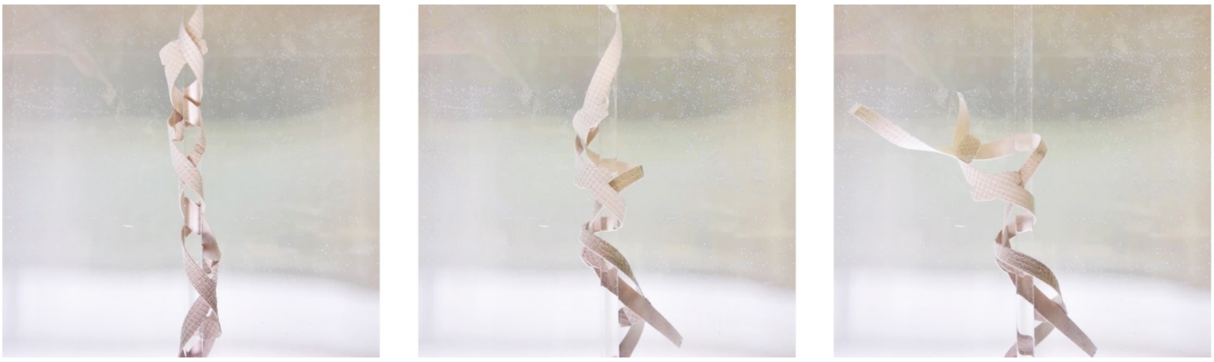

Movie S4. The same 4D-printed helical material system but with pocket mechanisms is initially stabilized on a 1.5 cm diameter support structure (it can also stabilize on a 3 cm diameter structure). After direct exposure to water, the system self-releases from its support and no longer resists sliding (video playback at 500x speed).

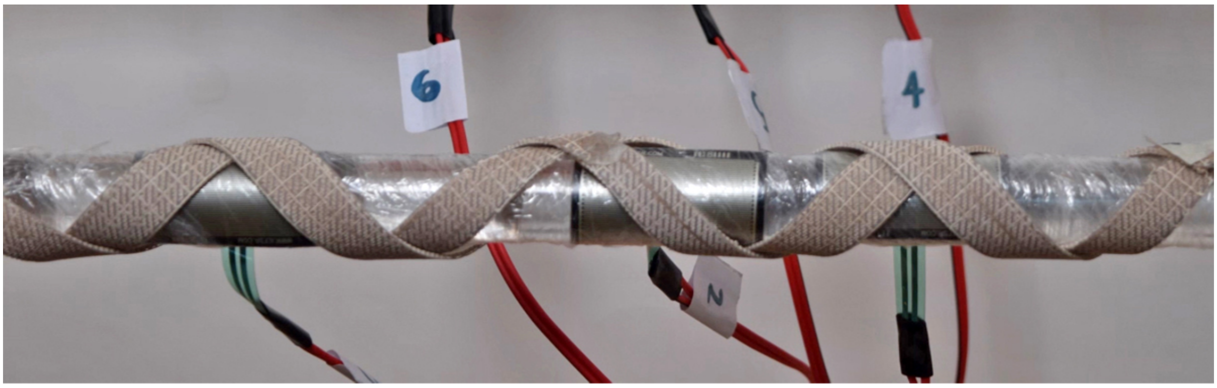

Movie S5. A 4D-printed helical material system self-tightening at 25-28% RH around a 3 cm diameter support (video playback at 1500x speed).

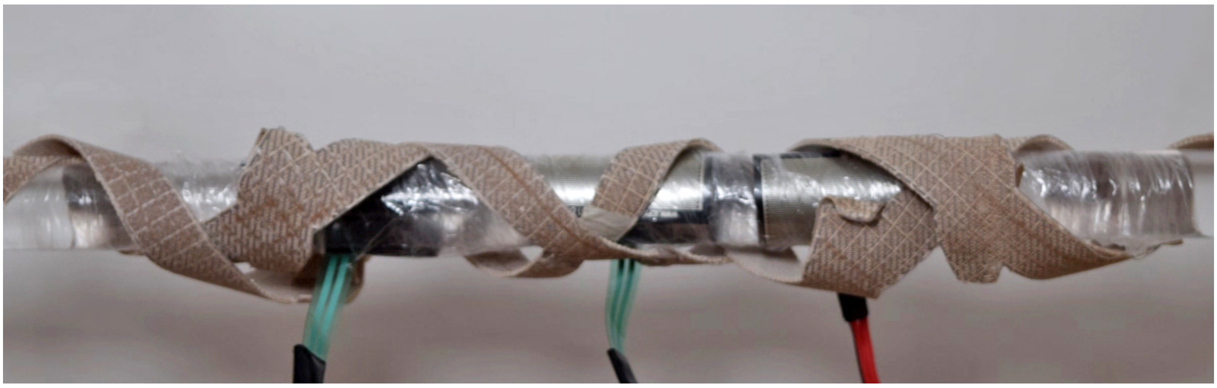

Movie S6. A 4D-printed helical material system with pocket mechanisms self-tightening at 25-28% RH around a 3 cm diameter support (video playback at 1500x speed).

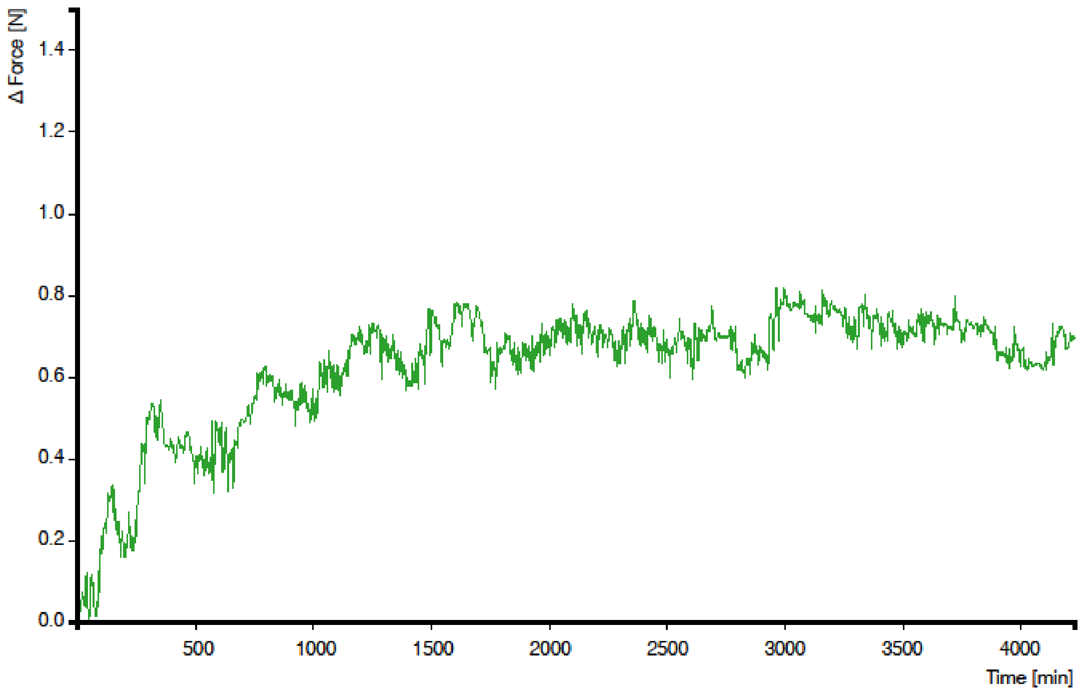

Figure S1. The change in grip force was measured in an extended experiment. An initially saturated (wet) 4D-printed helical material system with pocket mechanisms (spaced apart at 220 mm along the helix) was exposed to 25-28% RH conditions. The majority of forces are generated within the first 1080 minutes, after which the force generation plateaus over the course of 4200 minutes.

Table S1. All prototypes were printed using 1.75 mm diameter filaments according to the following setup and specifications, producing extruded paths with widths of 0.5 ± 0.075 mm.

FABRICATION SETTINGS				
Environment	27% - 30% R.H.		21° - 25° C	
Printer setup	Equipment: FELIX Tec 4 Dual Head		Bed temp: 45° C	
Actuating material	Material: WPC (Laywood-D3)	Nozzle: 0.35 mm Temp: 190° C	Feedrate: 700 mm/min	Flow: 0.033 mm of filament / mm
Restricting material	Material: ABS (MakerBot)	Nozzle: 0.35 mm Temp: 200° C	Feedrate: 1200 mm/min	Flow: 0.033 mm of filament / mm

Table S2. The relationship between mesostructure and motion is established through empirical studies. Displayed here is a catalogue of various specimen types and all of their material programming parameters for reproduction. The experimental data includes measurements collected immediately after printing, at high moisture content, and at low moisture content. At least 3 identical specimens were 4D-printed for each parameter study.

SPECIMEN (10 mm x 60 mm) S-0.4-2L				
Material Programming Parameters				
Actuating layer	Angle: 0°	Offset: 0.4 mm	Z offset: 0.2 mm	Layers: 2
Restricting layer	Angle: 90°	Offset: 1.5 mm	Z offset: 0.15 mm	Layers: 1

Averaged Measurements								
At 25% R.H.			Initial (as printed)			At 90% R.H.		
Weight: 0.344 g	Depth: 0.66 mm	Width: 10.8 mm	Weight: 0.356 g	Depth: 0.64 mm	Width: 11.0 mm	Weight: 0.408 g	Depth: 0.70 mm	Width: 11.2 mm

Change in weight:	Change in depth:	Change in width:	Min. bending diameter:
18.57%	4.77%	3.06%	65.26 mm

SPECIMEN (10 mm x 60 mm) S-0.5-2L				
Material Programming Parameters				
Actuating layer	Angle: 0°	Offset: 0.5 mm	Z offset: 0.2 mm	Layers: 2
Restricting layer	Angle: 90°	Offset: 1.5 mm	Z offset: 0.15 mm	Layers: 1

Averaged Measurements								
At 25% R.H.			Initial (as printed)			At 90% R.H.		
Weight: 0.294 g	Depth: 0.62 mm	Width: 10.8 mm	Weight: 0.308 g	Depth: 0.65 mm	Width: 10.9 mm	Weight: 0.368 g	Depth: 0.67 mm	Width: 11.0 mm

Change in weight:	Change in depth:	Change in width:	Min. bending diameter:
24.86%	7.84%	1.83%	62.79 mm

SPECIMEN (10 mm x 60 mm) S-0.5-3L				
Material Programming Parameters				
Actuating layer	Angle: 0°	Offset: 0.5 mm	Z offset: 0.2 mm	Layers: 3
Restricting layer	Angle: 90°	Offset: 1.5 mm	Z offset: 0.15 mm	Layers: 1

Averaged Measurements								
At 25% R.H.			Initial (as printed)			At 90% R.H.		
Weight: 0.396 g	Depth: 0.77 mm	Width: 10.8 mm	Weight: 0.415 g	Depth: 0.83 mm	Width: 10.9 mm	Weight: 0.488 g	Depth: 0.87 mm	Width: 11.0 mm

Change in weight:	Change in depth:	Change in width:	Min. bending diameter:
23.34%	11.85%	2.18%	63.15 mm

SPECIMEN (10 mm x 60 mm) S-0.5-4L				
Material Programming Parameters				
Actuating layer	Angle: 0°	Offset: 0.5 mm	Z offset: 0.2 mm	Layers: 4
Restricting layer	Angle: 90°	Offset: 1.5 mm	Z offset: 0.15 mm	Layers: 1

Averaged Measurements								
At 25% R.H.			Initial (as printed)			At 90% R.H.		
Weight: 0.506 g	Depth: 1.09 mm	Width: 10.8 mm	Weight: 0.522 g	Depth: 1.08 mm	Width: 10.8 mm	Weight: 0.661 g	Depth: 1.13 mm	Width: 11.0 mm

Change in weight:	Change in depth:	Change in width:	Min. bending diameter:
30.55%	3.35%	2.48%	122.06 mm

SPECIMEN (10 mm x 60 mm) S-0.6-2L

Material Programming Parameters

Actuating layer	Angle: 0°	Offset: 0.6 mm	Z offset: 0.2 mm	Layers: 2
Restricting layer	Angle: 90°	Offset: 1.5 mm	Z offset: 0.15 mm	Layers: 1

Averaged Measurements

At 25% R.H.			Initial (as printed)			At 90% R.H.		
Weight: 0.266 g	Depth: 0.62 mm	Width: 10.7 mm	Weight: 0.277 g	Depth: 0.67 mm	Width: 10.9 mm	Weight: 0.336 g	Depth: 0.72 mm	Width: 10.9 mm

Change in weight: 26.32%	Change in depth: 16.13%	Change in width: 2.34%	Min. bending diameter: 37.39 mm

SPECIMEN (10 mm x 60 mm) T-30-2L

Material Programming Parameters

Actuating layer	Angle: 30°	Offset: 0.5 mm	Z offset: 0.2 mm	Layers: 2
Restricting layer	Angle: 120°	Offset: 1.5 mm	Z offset: 0.15 mm	Layers: 1

Averaged Measurements

At 25% R.H.			Initial (as printed)			At 90% R.H.		
Weight: 0.284 g	Depth: 0.64 mm	Width: 10.6 mm	Weight: 0.322 g	Depth: 0.66 mm	Width: 10.9 mm	Weight: 0.341 g	Depth: 0.66 mm	Width: 10.6 mm

Change in weight: 19.95%	Change in depth: 2.59%	Change in width: 0.79%	Min. bending diameter: 32.81 mm

SPECIMEN (10 mm x 60 mm) T-45-2L

Material Programming Parameters

Actuating layer	Angle: 45°	Offset: 0.5 mm	Z offset: 0.2 mm	Layers: 2
Restricting layer	Angle: 135°	Offset: 1.5 mm	Z offset: 0.15 mm	Layers: 1

Averaged Measurements

At 25% R.H.			Initial (as printed)			At 90% R.H.		
Weight: 0.282 g	Depth: 0.67 mm	Width: 10.5 mm	Weight: 0.315 g	Depth: 0.73 mm	Width: 10.7 mm	Weight: 0.351 g	Depth: 0.71 mm	Width: 10.6 mm

Change in weight: 24.47%	Change in depth: 6.47%	Change in width: 0.67%	Min. bending diameter: 31.75 mm

SPECIMEN (10 mm x 80 mm) T-45-2L-Long

Material Programming Parameters

Actuating layer	Angle: 45°	Offset: 0.5 mm	Z offset: 0.2 mm	Layers: 2
Restricting layer	Angle: 135°	Offset: 1.5 mm	Z offset: 0.15 mm	Layers: 1

Averaged Measurements

At 25% R.H.			Initial (as printed)			At 90% R.H.		
Weight: 0.370 g	Depth: 0.73 mm	Width: 10.4 mm	Weight: 0.406 g	Depth: 0.68 mm	Width: 10.7 mm	Weight: 0.447 g	Depth: 0.74 mm	Width: 10.6 mm

Change in weight: 20.97%	Change in depth: 2.53%	Change in width: 1.71%	Min. bending diameter: 36.69 mm

SPECIMEN (15 mm x 60 mm) T-45-2L-Wide

Material Programming Parameters

Actuating layer	Angle: 45°	Offset: 0.5 mm	Z offset: 0.2 mm	Layers: 2
Restricting layer	Angle: 135°	Offset: 1.5 mm	Z offset: 0.15 mm	Layers: 1

Averaged Measurements

At 25% R.H.			Initial (as printed)			At 90% R.H.		
Weight: 0.427 g	Depth: 0.70 mm	Width: 15.3 mm	Weight: 0.454 g	Depth: 0.68 mm	Width: 15.7 mm	Weight: 0.454 g	Depth: 0.88 mm	Width: 15.5 mm

Change in weight: 6.45%	Change in depth: 25.24%	Change in width: 1.17%	Min. bending diameter: 30.69 mm

<table>
<tr><th colspan="9">SPECIMEN (10 mm x 60 mm) T-60-2L</th></tr>
<tr><th colspan="9">Material Programming Parameters</th></tr>
<tr><td>Actuating layer</td><td colspan="2">Angle: 45°</td><td colspan="2">Offset: 0.5 mm</td><td colspan="2">Z offset: 0.2 mm</td><td colspan="2">Layers: 2</td></tr>
<tr><td>Restricting layer</td><td colspan="2">Angle: 135°</td><td colspan="2">Offset: 1.5 mm</td><td colspan="2">Z offset: 0.15 mm</td><td colspan="2">Layers: 1</td></tr>
<tr><th colspan="9">Averaged Measurements</th></tr>
<tr><td colspan="3">At 25% R.H.</td><td colspan="3">Initial (as printed)</td><td colspan="3">At 90% R.H.</td></tr>
<tr><td>Weight:
0.287 g</td><td>Depth:
0.67 mm</td><td>Width:
10.5 mm</td><td>Weight:
0.333 g</td><td>Depth:
0.64 mm</td><td>Width:
10.8 mm</td><td>Weight:
0.333 g</td><td>Depth:
0.70 mm</td><td>Width:
10.7 mm</td></tr>
<tr><td colspan="2">Change in weight:
16.28%</td><td colspan="2">Change in depth:
3.98%</td><td colspan="2">Change in width:
1.26%</td><td colspan="3">Min. bending diameter:
61.38 mm</td></tr>
</table>
